# Supplementary material for: Patterns of Intron Gain and Loss in Fungi
Source: PLoS Biol. 2004 Nov 30;2(12):e422. doi: 10.1371/journal.pbio.0020422 (PMC532390; doi:10.1371/journal.pbio.0020422)
Supplement: Table S1 — Also available at http://genes.mit.edu/NielsenEtAl/. (4.3 MB ZIP). [file pbio.0020422.st001.zip › NielsenEtAl/html/234.html]

AN4817.1.NCU01968.1.MG10131.1.FG08672.1


```
 CLUSTAL W (1.82) Multiple Sequence Alignments - Introns Inserted


Sequence 1: NCU01968.1	590 aa
Sequence 2: MG10131.1	740 aa
Sequence 3: FG08672.1	743 aa
Sequence 4: AN4817.1	731 aa
Alignment Length: 787 aa
Number Identitical Residues: 243 aa
Alignment Score (without introns) 13791


MG10131.1 	-MAARDEAQPSFISRLPFFGGRKKNVPPRDSHESETDEAP--SNAPPKWSFGVLNDKKTI
NCU01968.1	------------------------------------------------------------
FG08672.1 	---MSENTNVPRWRRFLGAGAGSTAVPRQND-----------AYGPSKWSMGVLNDKETI
AN4817.1  	MAAAAGQPHEDMGSQQAEKGWWKRVFRSASSGNSRSSQGPEDIDYRPRATLGILSDRETD
          	  :   ..           .  .      .. .: :......    .  : .  .. .: 

MG10131.1 	EVPG1SVLLLADHKNEPLGLRNAPARTSHSSFPT--------------------------
NCU01968.1	----~-------------------------------------------------------
FG08672.1 	EVPG1SVLLLASHRNEPLGLRNVHARNSHSSIPTGFAVDERRASASSAAAAAAAASVAAE
AN4817.1  	EVPG1TVLLLSSNRNEPLGLRHQPQRTSASSLPVS-------------------------
          	. .. :    :.  ... .       .: :: . .                         

MG10131.1 	----ANFPEPHPPADVKKKTTDGKIILEPQPEDAPNDPLNWPKWRRDAALLSIGLYCMVG
NCU01968.1	------------------------------------------------------------
FG08672.1 	SHRPASSSRPASANEGKKKTSDGAIILEPQPEDSGNDPLNWPSWKRDAALLSLGFYCMIG
AN4817.1  	--HPPSRSSSRASAPPTKKTADGKIVLNPQPDDSRNDPLNWPVWRRDAALISLGFYCLMG
          	   ... . . ..   ...::..    ......: ... . .    .::  : .  .  .

MG10131.1 	GGQTSVLAAGFTDIARDYGVPVHSVSLTVG~LYMMGMGIGSVFASPTAILYGKRPVYLAS
NCU01968.1	------MAQRFR--------PALPGPLLHG1LYMLGMGLGSVIFSPTAILYGKRPVYLFG
FG08672.1 	GGTTPLIAAGFTDVAEEYDVDVERVALTTG~LYMMGMGLGSVVFSPTAILYGKRPVYLAS
AN4817.1  	GGMTPILAAGFNQVAEAYDVDTQKVAYTTG~LYMLGLGIGSVVMSPTAILWGKRPVYLLG
          	.. :. :*  * . :   .  .   .   * ***:*:*:***. ******:******* .

MG10131.1 	AILFIVTSVWCALSPSFPSLVAARVFQGIAVSPVECLPSATVAEIFFLHERAYRLGIYTL
NCU01968.1	ALLFVLTSVWCAVSKSFVSLVLGRIFQGIAVSPVECLPSATIAEIFFLHERAYRIGIYTL
FG08672.1 	AVMFIGTSLWSAWSPSFSSLLAARVFQGFAVAPVECLPSATIAEIFFLHERAYRIGIYTL
AN4817.1  	ATLFIISAVWCAVSPNYPSLVVARIFQGIAVSPVECLPSATIAEIYFLHERAYRVGIYTL
          	* :*: :::*.* * .: **: .*:***:**:*********:***:********:*****

MG10131.1 	LLLGGKNLIPLVSAAIIQSLSWRWVFW2VVTFVVAFCGVLLFLFVPETFWERVPHPKPKT
NCU01968.1	LLLGGKNLVPLVSAAVIESLGWRWTFW2VVAMVVGFCGFLLFLFVPETFWDRVPHPRKKS
FG08672.1 	LLLGGKNIVPLVSAVIIGSLGWRWTFW~ILAMIIGLGAVLLFLFVPETFWDRTPHARPKH
AN4817.1  	LLLGGKNLIPLVSAAIIGSLGWRWVFW~IVAIIVGACLVLIFFFLPETFWDRTPRPRRHR
          	*******::*****.:* **.***.** ::::::.   .*:*:*:*****:*.*:.: : 

MG10131.1 	P-SRPGLFKRISSR-YVHQHKPAERPATAHAAPTTTPS-EAASPDKRHDHHVMFAETTET
NCU01968.1	--SRPNFFRRMSSKQHIRRSDAAGVSVPPSALPSARHSEEGPTPAPAANHRRNVHVGFAT
FG08672.1 	PSKRPSFLRRLSSR-HQQVQRPAEEEKVLEPERKEHSSEASPTQDGRQSPAPSHHSHHQR
AN4817.1  	--KRPDFYRSVSDMVSHGLHR--GRTTNAHPRDISEDAPDAVSQKRSKKGHVGFIENEPE
          	  .**.: : :*.                 .      :  . :     .           

MG10131.1 	SSNNHGVEGSPEAKADDQHLGDSNKSEASETPSSE--DEKQRKPAMPSLDTQNVDREGAN
NCU01968.1	DTEEHHAQKDREAATRQEISEKSDELHDPSVPMTAGILAEDSHAARPELITQDPSSSDNS
FG08672.1 	ARNLHVGFAGPDAADKETTAPEVD-------------------SDSPKLLANSSDKDSAS
AN4817.1  	DEELEQEKQELPYGSDPVSLVQSQ-----------------SQVADPSVEPSDPESTEKP
          	  : .                . :                      *.: ... .     

MG10131.1 	QVPVSAHPRLESNTQSSPVVGGNVFAPMDPEKRQQQDKATAN--------VYSNHWRERE
NCU01968.1	TKPHVFYMPDNIEKPKSPYSS-ERFARQDHHETDTESQSVASSRAVGSRVPYTAALRDQP
FG08672.1 	--PSGADTATGSDSFNNPFKLGPSLENEKLDASDLEGPQKVK--------AYTHNLRQQP
AN4817.1  	----SLPPLTNGGATRSSDLEAGRFASPSPARSESADPAAGLSLP----VQYTNRLRDKT
          	                ..   .  :   .    :  .     : .      *:   *:: 

MG10131.1 	AKSFVQQLRPWHGRLNNDRWLKVAFRPFVLFSYPAVLWSSAMYACSVGWLIVVSEAVAVI
NCU01968.1	AKSFVQQLKPFHGRLNKDKWHKVAIRPLILFSYPAVLWSAIVYACSVGWLIVISESVAVI
FG08672.1 	PKSFTQTLKPWHGRLNGDSWFKVMIRPFVLYAYPAVLWSSAVYACSVGWLIVISESIAMI
AN4817.1  	KIPYTYYLRPWNGRISQDRWLRVAIRPFILFAYPAVLWSTAVYALSVGWLIVLSEVVSHI
          	  .:.  *:*::**:. * * :* :**::*::*******: :** *******:** :: *

MG10131.1 	YR-EKYYNFDALGVGLVYLSPFIGGVLGTAVAGKVSDIFVKAMSRRNGGLYEPEFRLIMA
NCU01968.1	YR-EGHYEFNALQTGLVYISPFVGGILGTAVAGKVSDVIVKAMARRNGGLYEPEFRLVMA
FG08672.1 	YRNSESYNFTALQTGLVYLSPFVGGILGTGVAGKVSDVIVKIMSRRNGGLYEPEFRLVMA
AN4817.1  	YQDRETYNFTALQTGLVYISPFVGGLLGTAVAGKISDIIVRYMTRRNGGVYEPEFRLIMA
          	*:.   *:* ** .****:***:**:***.****:**::*: *:*****:*******:**

MG10131.1 	LPIGITTVMGLMGFGWSAEERNNYMVPTVFFGIISFGCCLGSTTSITFCVDSYRQYAGEA
NCU01968.1	LPVAITTVIGLMGFGWSAQLGDHWIVPTVFFGVVSFGCSLGSTTSITFCVDSYRQYAGEA
FG08672.1 	IPILITTCIGLMGFGWSAEEKDHWMVPTFFFGVVSFGCSLGSTTAITFCVDSYRQYAGEA
AN4817.1  	IPIALSTTIGLMGFGWSAQEKDAWIVPTIFFGLVSFGCCLGSTTSITFCVDSYRQYAGEA
          	:*: ::* :*********:  : ::***.***::****.*****:***************

MG10131.1 	LVTLNFAKN~ILHGLVFSLFVTEWLEHDGAKSVFIWLGVIQLVVVAFAAPLFVYGKRCRM
NCU01968.1	LVTLNFSKN~IFHGLVFSLFVTGWLTDDGPKTVFIWIGIIQLILLFLTIPMYIYGKRARM
FG08672.1 	LVTLNFSKN~ILHGLVFSLFVTHWLHGEGPKMVYIWIGIIQLVLMLFTIPMYIYGKRARM
AN4817.1  	LVTLNWSKN1VFHGLVFSLFVVDWMEDDGARTVFIALGVIQFGLLLFSIPMYIFGKRARM
          	*****::** ::*********. *:  :*.: *:* :*:**: :: :: *::::***.**

MG10131.1 	WTVRNNFMEKF
NCU01968.1	WTVRKNFMEKF
FG08672.1 	WTVRANLMERF
AN4817.1  	WTVRKKLMERF
          	**** ::**:*
```
